# What is the genetic architecture of major psychiatric disorders?

**DOI:** 10.1192/j.eurpsy.2021.155

**Published:** 2021-08-13

**Authors:** W. Hennah, M. Gennarelli

**Affiliations:** 1 Neuroscience Center, University of Helsinki, Helsinki, Finland; 2 Department Of Molecular And Translational Medicine, University of Brescia, Brescia, Italy

**Keywords:** psychiatry, genomics, genetics

## Abstract

Taken as a whole, psychiatric disorders are considered as complex genetic disorders. There are clear genetic mutations and susceptibility factors to these disorders. However, these form the full spectrum of impact, frequency, and mutation type. With rare large scale chromosomal rearrangements and copy number mutations of high impact at one end, and common single nucleotide variations of minor impact at the other. This multitude of variation type also means that different epidemiological study designs are needed to test the genetic component of these disorders, from familial forms, to common population level studies. This process has been facilitated by advances in genomic analysis, that enable the measuring of genetic variation at a greater depth in a greater number of individuals and has led to a boom in genetic information. This has given us a greater understanding of the genetic aetiology of psychiatric disorders and how they are biologically related to each other. How this information can be translated to the clinics, can now be considered. Genetic testing in psychiatric disorders, is currently possible for certain disorders and mutation types, but is not universally advised. Much still remains to be understood about population level genetic risk factors before they could conceivably be utilised in the clinic. Whereas genetic testing of high impact mutations could be of use to the clinical programs, and are actively tested for in clinics across Europe.

**Disclosure:**

No significant relationships.

